# Risk for Hepatitis E Virus Transmission by Solvent/Detergent–Treated Plasma

**DOI:** 10.3201/eid2612.191482

**Published:** 2020-12

**Authors:** Pierre Gallian, Sébastien Lhomme, Pascal Morel, Sylvie Gross, Carole Mantovani, Lisette Hauser, Xavier Tinard, Elodie Pouchol, Rachid Djoudi, Azzedine Assal, Florence Abravanel, Jacques Izopet, Pierre Tiberghien

**Affiliations:** Aix-Marseille Université, Marseille, France (P. Gallian);; Institut National de la Santé et de la Recherche Médicale, Marseille (P. Gallian);; Etablissement Français du Sang, La Plaine, Saint-Denis, France (P. Gallian, P. Morel, S. Gross, E. Pouchol, R. Djoudi, P. Tiberghien);; Institut National de la Santé et de la Recherche Médicale, Toulouse, France (S. Lhomme, F. Abravanel, J. Izopet);; Hôpital Purpan, Toulouse (S. Lhomme, F. Abravanel, J. Izopet);; Etablissement Français du Sang Nouvelle Aquitaine, Bordeaux, France (C. Mantovani, A. Assal); Etablissement Français du Sang Ile-de-France, Ivry sur Seine, France (L. Hauser);; Etablissement Français du Sang Grand Est, Nancy, France (X. Tinard);; Université de Franche-Comté, Besançon, France (P. Tiberghien)

**Keywords:** hepatitis E virus, HEV, viruses, transmission, solvent/detergent–treated plasma, plasma, retrospective study, infectious dose, blood donors, zoonoses, France

## Abstract

Hepatitis E has emerged as a major transfusion-transmitted infectious risk. Two recipients of plasma from 2 lots (A and B) of pooled solvent/detergent–treated plasma were found to be infected by hepatitis E virus (HEV) that was determined to have been transmitted by the solvent/detergent–treated plasma. HEV RNA viral loads were 433 IU in lot A and 55 IU in lot B. Retrospective studies found that 100% (13/13) of evaluable lot A recipients versus 18% (3/17) of evaluable lot B recipients had been infected by HEV (p<0.001), albeit not necessarily at time of transfusion. Among evaluable recipients, 86% with a transfused HEV RNA load >50,000 IU were infected, most likely by the HEV-containing solvent/detergent–treated plasma, versus only 7% with a transfused HEV RNA load <50,000 IU (p<0.001). Overall, solvent/detergent–treated plasma might harbor HEV. Such an occurrence might result in a dose-dependent risk for transfusion-transmitted hepatitis E.

Hepatitis E virus (HEV) is a small, nonenveloped RNA virus belonging to the family *Hepeviridae*, genus *Orthohepevirus.* HEV genotypes 3 and 4 cause zoonotic infections described in countries in Europe and transmitted mostly by the fecal–oral route in contaminated food or the environment (*1*). Hepatitis E transmission by blood products has been reported, including plasma treated by pathogen-reduction methods (*2*,*3*).

Several studies have indicated that not all HEV-infected blood products cause infection in recipients, suggesting that blood products with a low residual plasma volume and provided by donors with low HEV viral loads might not be infectious (*4*,*5*). The lowest infectious dose resulting in proven or probable HEV transfusion-transmitted infection was 7,056 IU in a platelet concentrate (*6*), 31,600 IU in an erythrocyte concentrate (*4*), and 36,000 IU in a fresh frozen plasma (*7*).

In France, IgG seroprevalence studies indicate that HEV infection is widespread (*8*); high rates in the southern part of the country indicate that this region might be considered a hyperendemic area. Prevalence of HEV RNA in blood donors in France has been estimated to be 1 positive sample/750 donors–1 positive sample/2,218 donors (*9*,*10*). A total of 23 cases of transfusion-transmitted hepatitis with high imputability were reported during 2006–2016, including recipients of solvent/detergent–treated plasma (*3*).

Transfusion-transmitted hepatitis E involving solvent/detergent–treated plasma resulted in the identification of HEV-contaminated solvent/detergent–treated plasma lots, each providing plasma units for <350 recipients. We report results and lessons learned from the hemovigilance investigations after identification during 2012 of 2 HEV RNA–positive solvent/detergent–treated plasma lots.

## Materials and Methods

### Production of Solvent/Detergent–Treated Plasma

Until 2014, the French Transfusion Public Service (Etablissement Français du Sang) produced solvent/detergent–treated plasma that was manufactured from 100 apheresis plasma donations pooled in a volume of 70 L before being divided into a maximum of 350 individual units of 200 mL. Donations were qualified according to the French regulations. Interruption of solvent/detergent–treated plasma production by the Etablissement Français du Sang resulted from classification of this blood product as a pharmaceutical product according to European Union rules.

### HEV Molecular and Serologic Investigations

Samples from solvent/detergent–treated plasma and blood donations were tested for HEV RNA, and viral loads were estimated by using a reverse transcription PCR as described (*11*). Subsequently, genotypes and subtypes were characterized by sequencing and molecular comparison of strains in the open reading frame (ORF) 2/ORF3 genomic region (*12*) and in a fragment of ORF1 covering the polymerase gene (*2*). HEV IgG concentration in solvent/detergent–treated plasma lots and detection of HEV IgG detection in contributed plasma donations were measured by using an HEV IgG Enzyme Immunoassay Kit (Wantai Biologic Pharmacy Enterprise, http://www.ystwt.cn) as described (*13*).

### Hemovigilance Inquiries

All contributed plasma donations to a solvent/detergent–treated plasma batch found positive for HEV were tested to identify the involved plasma donor(s). Furthermore, an inquiry was conducted for all recipients transfused with plasma units from an HEV-contaminated solvent/detergent–treated plasma lot. Information collected for each recipient included initial manifestations and outcome, number of solvent/detergent–treated plasma units transfused, and, if available, results of molecular and serologic testing for HEV markers in archived pretransfusion samples and posttransfusion control samples.

## Results

### Transfusion-Transmitted Hepatitis E Index Case-Patients

Index case-patient 1 was a 50–59 year-old man who had a thrombotic microangiopathy treated by plasma exchange and was found to be infected by HEV in December 2011. Hepatitis E was associated with increased liver cytolysis (increased levels of aspartate aminotransferase and alanine aminotransferase) and resolved spontaneously. This patient had received 150 blood products: 70 solvent/detergent–treated plasma units, 78 Intercept (amotosalen + UVA)–treated plasma units (http://cerus.com), and 2 erythrocyte concentrates. Results for HEV RNA in blood were negative 2 days before transfusion of 2 solvent/detergent–treated plasma units (from lot A) and positive 45 days later. At that time, case-patient 1 was positive for HEV IgG HEV IgM. Investigations on archived samples showed that solvent/detergent–treated plasma was positive for HEV RNA (433 IU). 

Further investigations showed that 1 plasma donor who contributed blood to lot A was positive for HEV RNA (HEV-3f, 117,000 IU). All other donors to lot A were negative for HEV RNA. The concentration of HEV IgG in lot A was 0.35 IU/mL

Index case-patient 2 was a 50–59 year old man who was a liver transplant recipient (because of alcoholic cirrhosis) and found to be infected by HEV in October 2012, 3 months after transplantation. Chronic hepatitis E infection developed in the patient. This infection was successfully treated with ribavirin. This patient had received 61 blood products: 30 plasma units, among which 14 were from solvent/detergent–treated plasma (lot B), plus 25 erythrocyte concentrates and 6 platelet concentrates. In the context of liver cytolysis, a blood sample was positive for HEV RNA, IgG, and IgM. The patient was seronegative for HEV IgG and HEV IgM just before transfusion of the involved plasma. Further investigations showed that solvent/detergent–treated plasma from lot B was positive for HEV RNA, albeit with a low viral level (HEV-3f, 55 IU).

Further investigations showed that 1 blood donor (52-year-old man) who contributed to lot B was positive for HEV RNA (HEV-3f, 2,448 IU). All other donors to lot B were negative for HEV RNA. The concentration of HEV IgG in lot B was 1.13 IU/mL.

In both instances, molecular comparison of HEV-3f viral strains from the 2 patients and solvent/detergent–treated plasma lots indicated a 100% nucleic acid sequence homology in ORF1 and 2, thus establishing high imputability. Clinical manifestations and outcomes of both case-patients (recipients) have been documented elsewhere (*3*). Remaining, nontransfused solvent/detergent–treated plasma from both lots at time of notification were immediately quarantined and subsequently discarded.

### Hemovigilance Inquiry

A total of 557 solvent/detergent–treated plasma units of lot A (n = 270) or lot B (n = 287) had been transfused into 143 recipients (lot A 61, lot B 82). When recipients were transfused with several solvent/detergent–treated plasmas, all of them were from the same solvent/detergent–treated plasma lot (lot A or lot B). Medical staff in charge of all involved patients were notified. Among the 143 solvent/detergent–treated plasma recipients, 33.6% (n = 48; lot A 23, lot B 25) had died before investigations of causes related to their primary disease. A total of 21% were evaluable for viral markers (RNA and Ig type), including the 2 index cases (n = 30; lot A 13, lot B 17); results for the remaining 45.4% (n = 65; lot A 25, lot B 40) were not available. No clinical symptoms or biologic abnormalities suggestive of acute or chronic hepatitis E were reported, except for both index case-patients and 1 patient (r3) who were found to be infected by HEV before and after transfusion.

### Investigation of Evaluable Recipients

We provide results of hemovigilance follow-up for the 30 recipients (including the 2 index case-patients) for whom results were available (Figure). Intervals between the solvent/detergent–treated plasma transfusion and recipient assessment varied highly and ranged from 2 to 44 months after transfusion; there were no significant differences between lot A and lot B recipients. In addition to the 2 index case-patients, only 1 additional recipient was positive for HEV RNA 37 months after transfusion (lot A recipient). This recipient was a heart transplant recipient for whom an earlier blood sample obtained 1 month before transfusion of 5 solvent/detergent–treated plasma samples from lot A already harbored HEV RNA.

**Figure:**
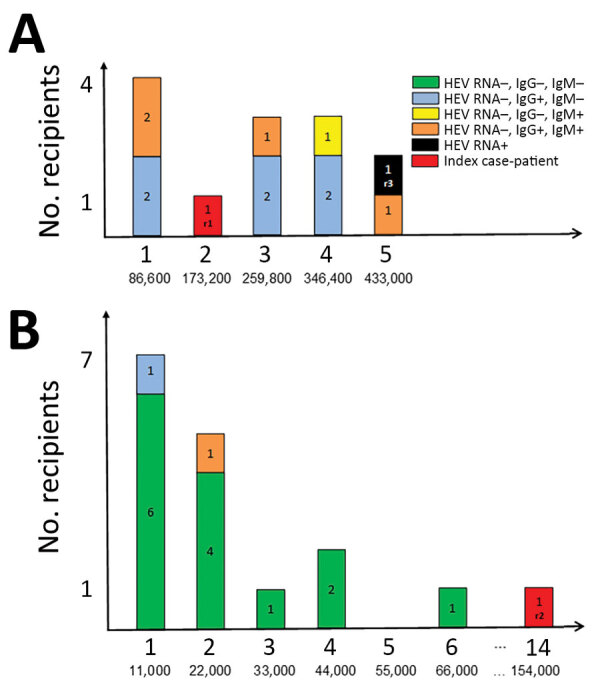
Transfused, HEV-infected solvent/detergent–treated plasma and recipient HEV status. A) Lot A; B) lot B. Top values along each x-axis indicate the number of solvent/detergent–treated plasma units transfused per recipient; bottom values indicate HEV viral load (IU/recipient). HEV, hepatitis E virus.

Among the remaining 27 case-patients, 1 recipient was HEV IgG negative and HEV IgM positive (assessed 14 months after transfusion), 5 were IgG positive and IgM positive (4–16 months after transfusion), 7 were IgG positive and IgM negative (2–44 months after transfusion), and 14 were IgG negative and IgM negative (4–31 months after transfusion). In addition to both index case-patients and the other HEV RNA–positive recipient mentioned, pretransfusion results were available for only 5 additional recipients at various times (range 1–21 months) before transfusion. Two were HEV IgG positive and IgM negative before and after transfusion, 1 was IgG negative and IgM negative before transfusion and IgG positive and IgM negative after transfusion, and the remaining 2 were IgG negative and IgM negative before and after transfusion. Both index case-patients (r1 and r2) received similar cumulative HEV viral loads (173,000 IU and 154,000 IU, respectively) (Figure). However, there was a major difference in the number of transfused solvent/detergent–treated plasma units: 2 (r1, lot A) and 14 (r2, lot B). Recipient 3 (r3, lot A) had received a higher viral load (433,000 IU) (Figure).

The frequency of HEV infection differed between recipients of solvent/detergent–treated plasma lots A and B. Although all (13/13, 100%) evaluable recipients of lot A solvent/detergent–treated plasma had been in contact with HEV (posttransfusion positive for HEV RNA or HEV IgG or IgM for all recipients), this finding was not observed for recipients of lot B, for which only 3 evaluable recipients had HEV antibody markers of infection (3/17, 18%) (p<0.001 by χ^2^ test). After exclusion of recipients who had proof of previous HEV infection (previously positive for HEV Ig or HEV RNA), the trend remained the same (10/10, 100% for lot A vs. 3/17, 18% for lot B). All other recipients who were HEV IgG positive and IgM negative (most of them in lot A) were tested >2 months after transfusion, thus potentially too late to detect IgM positivity after putative transfusion-transmitted hepatitis E.

To further evaluate the effect of solvent/detergent–treated plasma HEV RNA viral load on the risk for HEV infection in recipients, we clustered recipient data from both solvent/detergent–treated plasma lots and considered a 50,000 IU viral load threshold for infection, as suggested (*4*). We considered recipients positive for HEV RNA (n = 2 index case-patients) or positive for HEV IgG and HEV IgM (n = 5) (Figure) as most likely infected by a solvent/detergent–treated plasma transfusion and those negative for HEV RNA, HEV IgM, and IgG (n = 14) (Figure) as not infected by HEV. Other case-patients were considered not evaluable because non–transfusion-associated HEV infection had been demonstrated or could not be formally excluded. With such a threshold, a significant difference was observed between likely infected and noninfected recipients: 6/7 (86%) with a viral load >50,000 IU of HEV RNA were likely infected but only 1/14 (7%) with a viral load <50,000 IU were likely infected (odds ratio 12.0, 95% CI 1.77–81.3; p<0.001 by Fisher exact test).

## Discussion

We report results of investigations undertaken after identification of 2 solvent/detergent–treated plasma lots that contained HEV RNA. Such identification of solvent/detergent–treated plasma lots harboring HEV during December 2012 resulted in immediate introduction of HEV RNA screening of solvent/detergent–treated plasma lots, as well as plasma donors, and destruction of all remaining solvent/detergent–treated plasma units from lots that were positive for HEV RNA.

A portion of plasma units from these lots had been used before HEV RNA detection during December 2012. Efforts to test all involved recipients were only partially successful; 68% of evaluable patients remaining untested. Such a poor response rate, despite nationwide implementation of hemovigilance, highlights the difficulty of performing retrospective studies. Difficulties included lack of response by the medical staff in charge of the recipients despite several solicitations, unwillingness of recipients to undergo further biologic assessment, absence of recipient information, and no further investigations by the medical staff. For this last difficulty, medical staff often did not wish to take the risk for alarming the patient about a potential additional pathology that, if present, would not modify the course of their primary pathology. Furthermore, posttransfusion HEV testing, when undertaken, was most often performed in the absence of pretransfusion testing. Such posttransfusion HEV testing was performed several weeks to months after transfusion (i.e., after resolution of a putative HEV viremia and associated hepatitis E), thus preventing an accurate diagnosis of solvent/detergent–treated plasma transfusion-transmitted infections.

The optimal strategy for assessment of transfusion-transmitted HEV infection requires testing of molecular and serologic HEV markers at several time points before and after transfusion. Partial data (i.e., positive serologic results after transfusion only) and extended delay between informative samples are the main challenges in adequately documenting potential transfusion-transmitted HEV infection. This low frequency of evaluable recipients might have introduced a representative bias that was difficult to resolve precisely. However, one can expect that overall exposure to HEV was similar among nonevaluable recipients. Furthermore, recipients might have been exposed to a variety of infectious sources other than implicated plasma. However, because recipients who were given either lot were exposed similarly to other exposure risks, such occurrences should not greatly affect our findings and their interpretation.

Approximately 25% of the persons residing in France show seroreactivity against HEV (*8*). Accordingly, a recent study reported that HEV infection in transplant recipients resulted most often from sources of contamination other than transfusion (*14*). An additional difficulty is highlighted by the case-patient who was a heart transplant recipient (r3, lot A) and found to be infected by HEV after solvent/detergent–treated plasma transfusion but was also positive for HEV RNA 1 month before the transfusion, thus excluding, in principle, transfusion-transmitted hepatitis E. Investigations of recipients of lot B solvent/detergent–treated plasma showed that 14/17 recipients remained negative for HEV RNA, HEV IgG, and HEV IgM despite transfusion-mediated transmission of 11,000 IU–66,000 IU of HEV RNA (Figure, panel B). This finding could be explained by the low HEV viral level (55 IU) in each unit of lot B solvent/detergent–treated plasma, which might be too low to be infectious, or the presence of HEV IgG (1.13 IU/mL), which potentially provides complete or partial protection against HEV infection. However, the protective role of HEV IgG is controversial because a layer of lipid might encapsulate the virus and shield it from access to specific IgG, which would neutralize epitopes on the viral capsid (*15*,*16*). Conversely, none of the evaluable lot A recipients had a negative HEV IgG serologic status. Lot A plasma units contained a higher HEV RNA load and lower concentration of HEV IgG than lot B plasma units (433 IU vs. 55 IU of HEV RNA and 0.35 IU/mL vs. 1.13 IU/mL of HEV IgG).

These findings strongly suggest that at least a fraction of lot A plasma recipients, in addition to the index case-patient, had transfusion-transmitted hepatitis E that went clinically undetected. The threshold of 50,000 IU of HEV RNA in transfused plasma with regard to posttransfusion immunity against HEV (6/7 who had >50,000 IU vs. 1/14 who had <50,000 IU; p<0.001) further suggests that a large fraction of the seropositive recipients was infected at time of transfusion and that the infectious risk is proportionate to the transfused viral load. Alternatively, difference in frequency of seropositivity between recipients of solvent/detergent–treated plasma lot A versus lot B could be caused by differential geographic nontransfusion HEV infection (*8*). However, widespread issuing of the lot A and lot B solvent/detergent–treated plasma throughout France, in addition to the magnitude of the difference in HEV seroprevalence between recipients of both lots, make this hypothesis unlikely. A threshold of 50,000 IU of HEV RNA for plasma-mediated hepatitis E approaches the 3–4 log IU threshold reported elsewhere for plasma (*4*,*7*).

The lowest reported HEV RNA dose associated with transfusion-transmitted hepatitis E by fresh frozen plasma is 36,000 IU of HEV RNA (*7*). We observed 3 lot B solvent/detergent–treated plasma transfusion recipients who received serial transfusions with cumulative viral loads >36,000 IU of HEV RNA and who nevertheless did not seroconvert after transfusion. This finding might be partly related to a protective effect of the concurrent presence of HEV IgG in solvent/detergent–treated plasma. Overall, the minimal HEV RNA viral load in blood products needed to cause transfusion-transmitted hepatitis E might ultimately be difficult to determine. Risk for transfusion-transmitted hepatitis E might also depend on a combination of additional factors, such as concentration of HEV antibodies in the blood product, recipient immune competence, more specifically immune status with regard to HEV, and viral genotypes and subtypes.

In conclusion, solvent/detergent–treated plasma transfusion technology does not prevent transfusion-transmitted hepatitis E, as can be expected with nonenveloped viruses, such as HEV, hepatitis A virus, and parvovirus B19 (*17*). Plasma donation pooling, most often undertaken when producing solvent/detergent–treated plasma for transfusion, increases the risk for transfusion-transmitted hepatitis E, despite the viral level reduction associated with pooling and the putative protective effect of the low concentration of HEV IgG provided by donors who have resolved their infection. Such transfusion-transmitted hepatitis E might not be diagnosed. HEV testing of solvent/detergent–treated plasma transfusion in regions to which HEV is endemic is now mandatory according to the European pharmacopoeia (*18*). Overall, our observations highlight infectious risks associated with blood donation pooling when an infectious agent goes undetected and is resistant to an applied pathogen reduction technology.
